# Validation of asynchronous quantitative bone densitometry of the spine: Accuracy, short-term reproducibility, and a comparison with conventional quantitative computed tomography

**DOI:** 10.1038/s41598-017-06608-y

**Published:** 2017-07-24

**Authors:** Ling Wang, Yongbin Su, Qianqian Wang, Yangyang Duanmu, Minghui Yang, Chen Yi, Xiaoguang Cheng

**Affiliations:** 1 Department of Radiology, Peking University Fourth School of Clinical Medicine, Beijing Jishuitan Hospital, Xicheng District, Beijing, China; 2 Department of Epidemiology, Peking University Fourth School of Clinical Medicine, Beijing Jishuitan Hospital, Xicheng District, Beijing, China; 3 Department of Radiology, Forth Affiliated Hospital of Anhui Medical University, Anhui, China; 4 Department of Traumatology and Orthopedic Surgery, Peking University Fourth School of Clinical Medicine, Beijing Jishuitan Hospital, Xicheng District, Beijing, China

## Abstract

Asynchronous calibration quantitative computed tomography (QCT) is a new tool that allows the quantification of bone mineral density (BMD) without the use of a calibration phantom during scanning; however, this tool is not fully validated for clinical use. We used the European spine phantom (ESP) with repositioning during scanning and assessed the accuracy and short-term reproducibility of asynchronous QCT. Intra-scanner and intra-observer precision were each calculated as the root mean square of the standard deviation (RMSSD) and the coefficient of variation (CV-RMSSD). We also compared asynchronous and conventional QCT results in 50 clinical subjects. The accuracy of asynchronous QCT for three ESP vertebrae ranged from 1.4–6.7%, whereas intra-scanner precision for these vertebrae ranged from 0.53–0.91 mg/cc. Asynchronous QCT was most precise for a trabecular BMD of 100 mg/cc (CV-RMSSD = 0.2%). For intra-observer variability, overall precision error was smaller than 3%. In clinical subjects there was excellent agreement between the two calibration methods with correlation coefficients ranging from 0.96–0.99. A Bland–Altman analysis demonstrated that methodological differences depended on the magnitude of the BMD variable. Our findings indicate that the asynchronous QCT has good accuracy and precision for assessing trabecular BMD in the spine.

## Introduction

Bone mineral density is a surrogate indicator of bone strength that plays an important role in the management of osteoporosis and related fractures^1, 2^. Different from areal bone mineral density computed by dual-energy X-ray absorptiometry (DXA), bone mineral density (BMD) derived from quantitative computed tomography (QCT) is a volumetric measure of the vertebral trabecular bone. Given the high turnover rate of trabecular bone compared to cortical bone, BMD calculated from QCT offers substantially higher sensitivity. Yet, radiation doses associated with CT limit the application of QCT in osteoporosis screening.

Recently, asynchronous calibration QCT was described as a new tool for quantifying BMD^3^. Asynchronous calibration means that a calibration phantom is not necessary during QCT scanning. Rather, asynchronous QCT utilizes phantom data obtained separately from CT scans to calibrate data in Hounsfield units for the measurement of BMD. This approach is convenient for the assessment of BMD during routine abdominal and/or lung CT scans, and for the identification of patients who have an increased risk of fracture with diagnostic CT scanning. The 2015 International Society for Clinical Densitometry (ISCD) official position states that the in-scan calibration phantom for density-based QCT measurement can be replaced with asynchronous calibration if scanner stability is maintained^4, 5^. Opportunistic screening enabled by the use of asynchronous calibration may therefore improve the current understanding of bone health status and decrease the number of undiagnosed or overlooked cases of osteoporosis.

Few studies to date have compared conventional QCT, asynchronous QCT, and DXA^3, 6–8^. Moreover, asynchronous QCT has not yet been fully validated for the clinical measurement of spinal BMD. Although an early study by Brown *et al*. reported the precision of asynchronous QCT using the Mindways QA phantom, this study did not perform an accuracy assessment using the European spine phantom (ESP), which is a standard evaluation tool for bone densitometry. Thus, we performed 10 ESP scans with repositioning in order to assess the accuracy and short-term reproducibility of asynchronous quantitative bone densitometry, and to compare spine BMD results of asynchronous and conventional QCT in 50 clinical subjects.

## Methods

### Evaluation of accuracy and short-term reproducibility with ESP

**Figure 1:**
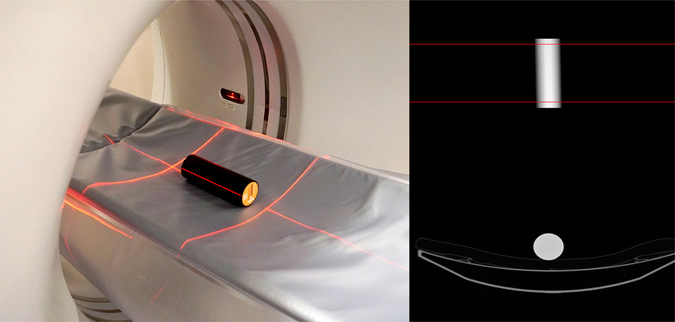
Quality assurance (QA) for asynchronous quantitative computed tomography using the Model 4 asynchronous QA phantom.

The ESP (QRM, Erlangen, Germany) was recommended by the International DXA Standardization Committee as a possible standard for use in DXA as well as QCT^9^. The ESP consists of three simulated vertebrae that are designed to give trabecular density values of 50, 100, and 200 mg/cm^3^ of hydroxyapatite, respectively. To simulate slight differences seen on daily quality control (QC) charts due to repositioning of the phantom, we performed each phantom scan with repositioning of the ESP. This method was recommended in the 2005 ISCD Official Position and reported to improve the estimate of the mean measure by a factor of nearly three^10^. Briefly, the ESP was placed on the scanner table on top of the table pad and aligned along the long axis of the table. CT scanning parameters were summarized in Table 1. Images were transferred to a QCT workstation and analysed using the 3D spine function version 5.10 of Mindways QCT pro software (Mindways Software Inc., Austin, TX, USA). This novel version includes a conventional QCT module and an asynchronous calibration module. For asynchronous QCT measurements, a new Model 4 asynchronous calibration phantom (Mindways Software Inc.) (Fig. 1) was scanned for quality assurance (QA) calibration. A Model 3 conventional calibration phantom (Mindways Software Inc.) was used to validate QA calibration for conventional QCT analysis. To evaluate short-term intra-scanner precision, a ten-scan series was performed in duplicate over two sessions, with one month between each scanning session. For the evaluation and comparison of asynchronous and conventional QCT, a Toshiba Aquilion 80-slices CT scanner (Toshiba Medical Systems Corp., Tokyo, Japan) was used to scan the ESP (no. 145) in the presence and absence of a Mindways calibration phantom, ten times each. Each group of ten scans was analysed individually and the average of each parameter was used to compute accuracy. To further compare of the effect of inter-scanner differences, The ESP was also scanned in 16-slice CT scanner for ten times with a calibration phantom. Based on the BMD measured using conventional QCT on each scanner in the different ESP regions, the interchangeability of the two CT systems was showed. Scan parameters of the two CT scanners were summarized on Table 1.

**Table 1:** Computed tomography (CT) scan parameters for phantom and patient image acquisition.

	European spine phantom	Patient spine
Scanner type	80-slice Toshiba Aquilion	16-slice Toshiba Aquilion
Voltage (kV)	120	120
Exposure (mAs)	100	Auto exposure
Reconstruction kernel	Standard	Standard
DFOV (mm)	400	400
Slice thickness (mm)	1.0	1.0
Table height (cm)	120	90

DFOV, display field of view.

### Subjects and clinical data

Fifty subjects (25 female subjects and 25 male subjects) were retrospectively enrolled in August 2016, one week before the date of Model4 Quality Assurance Scanning, from the emergency room radiology department of Beijing Jishuitan Hospital. In our emergency room radiology department, there are 10 to 20 clinical spine QCT scans on each day. For inclusion, eligible patients had to have conventional spine QCT BMD examinations within one week to avoid uncertainty regarding calibration stability in retrospective measurements. The exclusion criteria included fixation of the spine affecting image quality and more than two vertebrae compressive fractures. The 3D spine function version 5.10 of Mindways QCT pro software (Mindways Software Inc., Austin, TX, USA) was used to perform L1–L3 BMD analysis of all 50 cases, once with the conventional QCT module and once with the asynchronous calibration module. To further assess intra-observer precision, all 50 cases were analysed with the asynchronous QCT module two times by one radiologist, with one week between each analysis. This study was approved by the Ethics Committee of Beijing Jishuitan Hospital, Xicheng District, Beijing, China, and all methods were performed in accordance with the approved guidelines, and all the subjects signed informed consents.

### Image acquisition

For ESP, QCT scans were acquired using a Toshiba Aquilion 80-slice CT scanner (Toshiba Medical Systems Corp., Tokyo, Japan) with and without a calibration phantom (Mindways Software Inc., Austin, TX, USA). For clinical assessments, spine QCT scans were acquired using a Toshiba Aquilion 16-slice CT scanner (Toshiba Medical Systems Corp., Tokyo, Japan) with a calibration phantom (Mindways Software Inc., Austin, TX, USA). As the clinical scans were retrospectively from patients who aimed to have conventional QCT examinations, in our department the clinical QCT scans were all performed by 16-slice CT scanner in the emergency room radiology department. The reason for performing ESP scans on 80-slice CT scanner is that we want to investigate the intra-scanner precision on another scanner to show the equivalence. Scan parameters for both CT scanners were as follows: 120 kVp, 125 mAs, 1-mm slice thickness, 50-cm scan field of view, and a matrix size of 512 × 512 in spiral and standard reconstructions.

### Statistics analysis

The accuracies of conventional and asynchronous QCT for ESP scans were assessed by calculating the average of percentages of difference between measured values and expected values given by the manufacturer. Mann-Whitney U tests were used to compare values acquired using the two methods and to compare the interchangeability of the two CT scanners systems. A linear regression analysis was used to compare the density values acquired using the two methods, and a Bland–Altman analysis was conducted to assess differences between the conventional and asynchronous QCT measurements. As per the ISCD recommendation, short-term precision (intra-observer and intra-scanner precision) was calculated in terms of RMSSD and CV-RMSSD values.

### Data Availability

The datasets generated during and/or analyzed during the current study are available from the corresponding author on reasonable request.

## Results

### Accuracy

**Figure 2:**
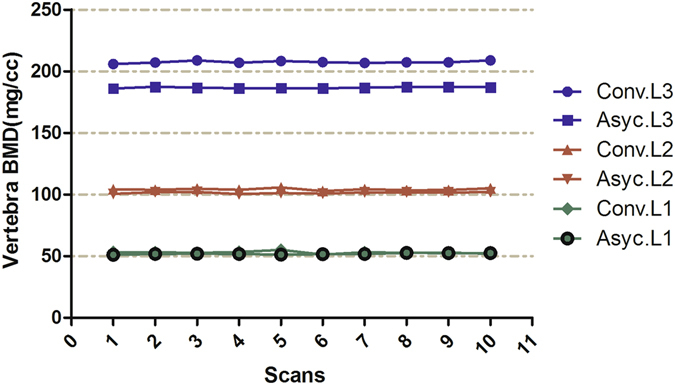
Ten-scan results of asynchronous and conventional quantitative computed tomography (QCT) for ESP study. Asyc., asynchronous QCT method; Conv., conventional QCT method.

The results of the accuracy analysis based on 10 ESP scans with repositioning are summarized in Table 2. The accuracies of conventional and asynchronous QCT were different, ranging from 3.7–5.9% for conventional QCT and from 1.4–6.7% for asynchronous QCT. Asynchronous QCT had higher accuracy than conventional QCT for measuring trabecular BMD in 50 and 100 mg/cc ESP (L1 and L2, respectively) vertebrae, whereas conventional QCT showed better accuracy for measuring the 200 mg/cc ESP L3 vertebra. Conventional QCT tended to overestimate ESP trabecular bone, whereas asynchronous QCT underestimated the BMD of the ESP L3 vertebra. The mean trabecular volumetric BMD (vBMD) for all three ESP vertebrae using conventional QCT was significantly higher than that obtained using asynchronous QCT. Figure 2 shows the results of ten scans performed with asynchronous QCT and conventional QCT; consistency was excellent for both methods.

**Table 2:** The accuracies of asynchronous and conventional quantitative computed tomography (QCT) and comparisons of inter-scanner differences.

	Accuracies of asynchronous and conventional QCT using 80-slice CT scanner							Inter-scanner differences		
Site	ESP BMD (mg/cm^3^)	Conv. values (mg/cm^3^)	RE	Asyc. values (mg/cm^3^)	RE	Conv. − Asyc. (mg/cm^3^)	P-value	Conv. values on 16-slice scanner (mg/cm^3^)	Diff. (mg/cm^3^)	P-value
L1	50	52.94 ± 0.99	5.9%	51.89 ± 0.53	3.8%	1.05	<0.0001	52.14 ± 1.45	0.80	0.66
L2	100	104.18 ± 0.85	4.2%	101.40 ± 0.61	1.4%	2.78	<0.0001	105.00 ± 0.37	−0.82	0.37
L3	200	207.40 ± 0.97	3.7%	186.70 ± 0.53	6.7%	20.71	0.0029	204.85 ± 1.35	2.55	0.023

Asyc., asynchronous QCT method; BMD, bone mineral density; Conv., conventional QCT; ESP, European spine phantom; RE, Relative error; Diff. difference defined as 80-slice scanner – 16-slice scanner. Data represent the mean ± standard deviation.

### Intra-scanner short-term reproducibility

The intra-scanner precision of asynchronous QCT for vBMD across all three vertebrae ranged from 0.53–0.91 mg/cc. Asynchronous QCT was most precise (in terms of the root mean square of the standard deviation [RMSSD]) for the 100 mg/cc ESP L2 vertebra with a coefficient of variance (CV)-RMSSD value of 0.2%.

### Clinical study

Table 3 shows that among 25 male subjects and 25 female subjects, the mean age was 62 ± 9 years (range, 33–85 years), the mean height was 165.9 ± 7.9 cm (range, 150.0–181.0 cm), the mean weight was 70.7 ± 9.8 kg (range, 50.0–89.5 kg), and the mean BMI was 25.6 ± 2.7 kg/m^2^ (range, 20.4–32.8 kg/m^2^). Of the 50 subjects, twelve female subjects and 10 male subjects had osteoporosis, and two patients had other vertebrae compressive fractures.

**Table 3:** Characteristics of clinical subjects with QCT scans acquired using a 16-slice CT scanner.

	Male subjects (n = 25)	Female subjects (n = 25)	Total subjects (n = 50)
Age (years)	62 ± 11	62 ± 8	62 ± 9
Weight (kg)	76.1 ± 7.0	65.1 ± 9.1	70.7 ± 9.8
Height (cm)	170.6 ± 5.7	161.0 ± 6.0	165.9 ± 7.9
BMI (kg/m^2^)	26.2 ± 1.9	25.1 ± 3.3	25.6 ± 2.7
Osteoporosis	10/25	12/25	22/50

BMI, body mass index. Data represent the mean ± standard deviation.

### Relationships between conventional and asynchronous QCT scanning results

**Figure 3:**
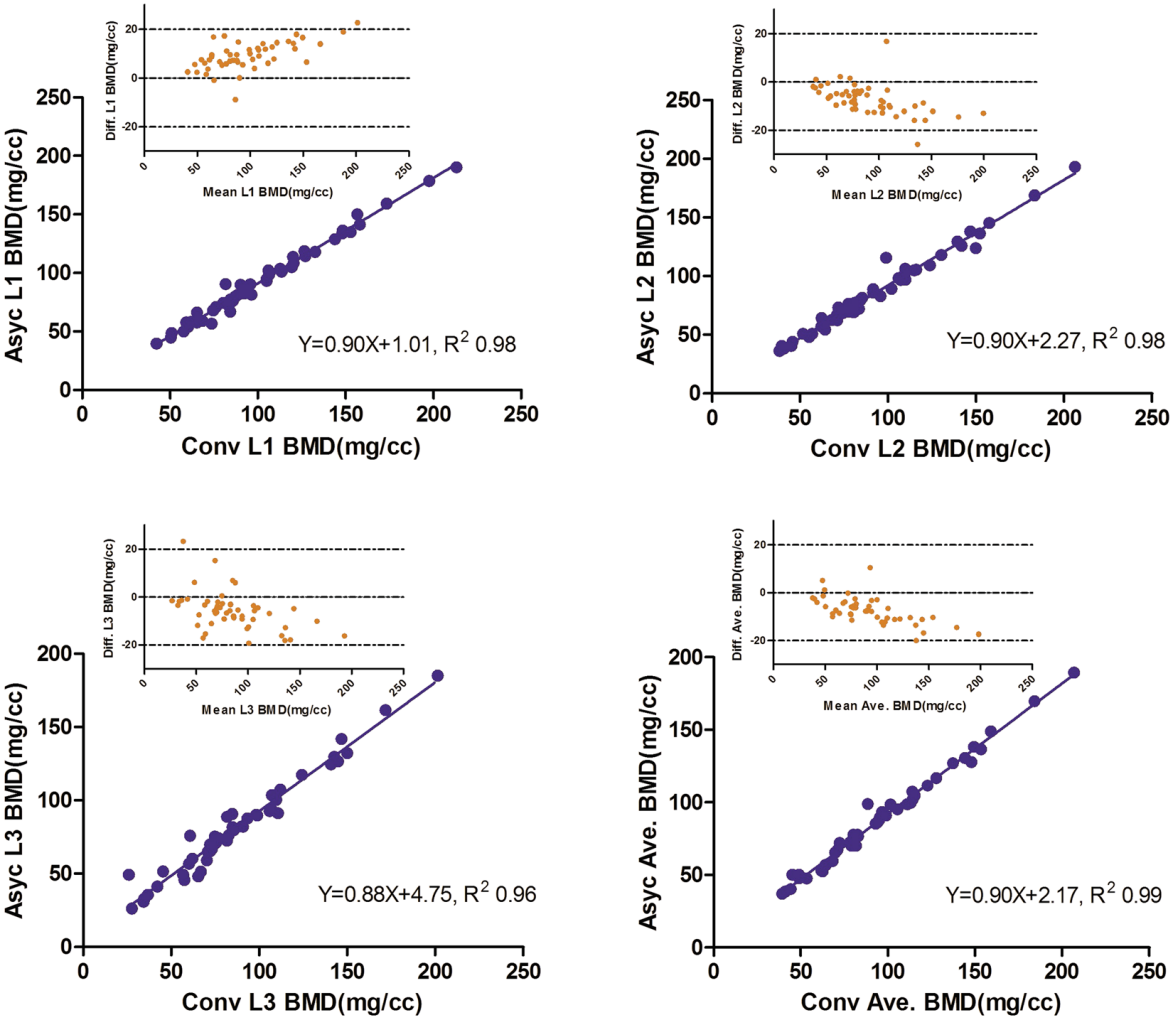
Correlation scatter plots for conventional and asynchronous quantitative computed tomography (QCT) outcomes of clinical subjects and Bland–Altman plots (small upper panel inserts) for conventional and asynchronous QCT BMD measurements of clinical subjects. Small upper inserts: Y-axis (Diff) is defined as (Asyc. −Conv.); X-axis is defined as the mean value of (Asyc. +Conv.).

Figure 3 shows correlations between the results of conventional and asynchronous QCT for each vertebra (L1–L3) and the average of all 3 vertebrae of the clinical subjects. The analysis demonstrated excellent agreement between the two methods, with correlation coefficients (R^2^) ranging from 0.96–0.99. A Bland–Altman analysis (small upper panel inserts in Fig. 3) showed differences between conventional and asynchronous QCT for different vertebrae with respect to a linear correlation. Methodological differences (expressed as absolute differences) depended on the magnitude of the BMD variable.

### Intra-observer variability

Table 4 summarizes the RMMS and CV-RMSS values for repeated measures with the asynchronous calibration method. The intra-observer precision for asynchronous QCT measured across all three ESP vertebrae ranged from 2.68–3.80 mg/cc. Overall precision error for BMD was smaller than 3%, consistent with the known precision of QCT technology.

**Table 4:** Intra-observer and intra-scanner reproducibility of asynchronous quantitative computed tomography (QCT).

Intra-observer variability					Intra-scanner variability				
Subjects (n = 50)	Obs. 1	Obs. 2	RMSSD	CV-RMSSD	ESP scans (n = 10)	Scan 1	Scan 2	RMSSD	CV-RMSSD
L1 vBMD	93.04 ± 34.58	94.02 ± 35.14	3.12	2.5	L1 vBMD	51.89 ± 0.53	51.79 ± 0.20	0.56	0.7
L2 vBMD	86.76 ± 34.01	87.64 ± 34.43	3.73	2.6	L2 vBMD	101.40 ± 0.61	101.67 ± 0.40	0.91	0.6
L3 vBMD	81.44 ± 33.42	81.39 ± 33.51	2.68	2.2	L3 vBMD	186.70 ± 0.53	186.55 ± 0.46	0.53	0.2

CV, coefficient of variation; Obs., observation; RRMSD, root mean square of the standard deviation; vBMD, volumetric bone mineral density. Data represent the mean ± standard deviation.

## Discussion

In this study, we determined the accuracy and intra-operator precision of asynchronous QCT using an ESP vertebrae dataset and data retrospectively collected from clinical subjects. In both datasets, the precision of BMD measurement was around 3%. Our results suggest that vertebral trabecular BMD measurements can be conveniently obtained by asynchronous QCT with good accuracy and precision.

The results of an analysis based on ten ESP scans demonstrated that asynchronous QCT had excellent accuracy for measuring trabecular BMD and agreed well with conventional QCT. Asynchronous QCT had slightly higher accuracy than conventional QCT for measuring trabecular BMD in 50 and 100 mg/cc ESP vertebrae; however, the differences between the two methods for 50 and 100 mg/cc vertebrae were relatively small (1.05 mg/cc and 2.78 mg/cc, respectively). In contrast, conventional QCT was more accurate for measuring the 200 mg/cc ESP vertebra, suggesting that the asynchronous QCT method may underestimate the BMD of high-density bone. While traditional QCT utilizes a calibration equation obtained from each individual slice, asynchronous QCT uses the same calibration equation for all data. This may result in lower variability in high-BMD bone for asynchronously calibrated results. Inconsistent with our finding, Brown *et al*. showed excellent agreement between conventional and asynchronous QCT using high-BMD Mindways QA phantom scan data^3^. The reason for this discrepancy is unclear; however, one explanation may be related to the fact that peak histogram values used in the calibration procedure depend on noise and thus exposure settings, the reconstruction kernel, and slice thickness^5^.

True *in vivo* precision measurements were obtained by measuring subjects twice at a one-month interval with repositioning. Repositioning has a lower impact on precision in three-dimensional QCT than in traditional two-dimensional slice-based imaging, where the location of the slice relative to the vertebral body is determined at the time of acquisition using the scout view taken before the actual CT scan. In an earlier study, Brown *et al*.^3^ investigated inter-observer variability for the asynchronous method using data from 43 patients aged 63.8 ± 8.6 years and reported RMSSD and CV-RMSSD values of 4.34 mg/cc and 3.67%, respectively; bias was not considered to be clinically important in the context of osteoporosis screening. In our study, we assessed the intra-observer reproducibility for asynchronous QCT and calculated RMSSD values of 3.12, 3.73, and 2.68 mg/cc and CV-RMSSD values of 2.5%, 2.6%, and 2.2% for the ESP L1–L3 vertebrae, respectively. Inter-observer reproducibility for asynchronous QCT reported by Brown *et al*. is therefore similar to the calculated intra-observer variability in our study.

In one phantomless QCT BMD study, inter-observer variability was 3.1 mg/cc and CV-RMSSD was 4.0%^11^, which are high compared to the results of our study. Although previous reports have indicated that non-calibrated Hounsfield unit values from CT scanners may be used for the opportunistic screening of low bone mass^11–13^, the use of a phantom calibration standard guarantees that the derived BMD computations will be consistent across CT scanners from different manufacturers and consistent across different scanning X-ray energy levels.

Lastly, we found that asynchronous and synchronous QCT results were highly correlated; regression lines for each method were not significantly different. Accordingly, it appears that bias (expressed as the absolute difference between method results) depends on the magnitude of the BMD variable. Different from conventional QCT utilizing a calibration equation obtained from each individual slice, the asynchronous QCT uses the same calibration equation for all slices and could have such calibration before or after CT images acquisition. The different calibration methods might be one of important causes of the bias. Further, there might be a phantom-induced bias between asynchronous and conventional QCT. Brown *et al*. had investigated the phantom-induced bias on clinical individuals and found that the bias induced by the presence of the phantom was 2.3 mg/cm3 when asynchronous calibration was applied^3^.

The present study had several limitations that most notably affected our precision assessments. First, due to concerns about radiation doses, it was not appropriate to scan patients twice in one session, so we performed ESP scans to investigate intra-scanner bias; however, this approach does not meet conservative recommendations (>27 degree of freedom, DOF) and may have underestimated precision error^14^. Second, we did not analyse the long-term precision of asynchronous QCT; this should be investigated in future studies. Lastly, the BMD of volunteers recruited in precision studies is often normal. In this study, we selected patients from a population with low-to-normal BMD. Given that BMD typically decreases with age, precision errors are typically larger in elderly individuals^15^. Additionally, precision errors are typically estimated by scanning a subject twice on the same day with repositioning in between scans and all scans performed by the same operator. Instead, we report precision using repeat scans taken at a one-month interval. We believe that these aspects of our study provide a more accurate reflection of the real precision of asynchronous QCT measurement. Further studies are necessary to confirm and extend our results.

In conclusion, we demonstrate that asynchronous QCT could be used for spine BMD screening based on the results of accuracy assessment for volumetric trabecular BMD in the spine and good short-term precision; however, even when the asynchronous and synchronous methods are highly correlated, the presence of bias was observed.
